# The MD-PhD program in Geneva: a 10-year analysis of graduate demographics and outcomes

**DOI:** 10.1186/s12909-020-02364-2

**Published:** 2020-11-12

**Authors:** Andre Dos Santos Rocha, Cristophe Combescure, Francesco Negro

**Affiliations:** 1 Unit for Anaesthesiological Investigations, Department of Acute Medicine, University of Geneva, Rue Michel-Servet 1, 1206 Geneva, Switzerland; 2 European MD-PhD Association, Groningen, The Netherlands; 3 Division of Clinical Epidemiology, University Hospitals of Geneva, Geneva, Switzerland; 4 MD-PhD Committee, Faculty of Medicine, University of Geneva, Geneva, Switzerland; 5 Divisions of Gastroenterology and Hepatology and of Clinical Pathology, University Hospitals of Geneva, Geneva, Switzerland

**Keywords:** Physician-scientist, MD-PhD, Medical research, Clinical research

## Abstract

**Background:**

MD-PhD programs confer degrees that empower medical doctors with in-depth scientific skills to contribute to biomedical research and academic medicine, alongside clinical practice. Whilst the career options and research opportunities related to graduates following these programs in the US are well documented, little is known about their European counterparts. In this article, we studied graduates who had completed the MD-PhD program at the University of Geneva between 2010 and 2019.

**Methods:**

A cross-sectional survey was performed in April 2019, targeting all medical doctors who had obtained the MD-PhD degree from the University of Geneva since 2010. Demographics, opinions, and career outcomes of the MD-PhD graduates were assessed through an online anonymous questionnaire.

**Results:**

Twenty-one questionnaires were collected from 31 MD-PhD graduates (response rate 65.5%). Most respondents (57.1%) had performed an MD-PhD training in basic sciences; however, only 14.3% had pursued this type of research thereafter. Most of the respondents held a position at a University hospital (90.5%), although a significant number of them were no longer involved in research in their current position (28.6%). 85.7% mentioned obstacles and challenges in combining clinical duties with research. Despite this, the majority (85.7%) declared that the MD-PhD degree had given them advantages in their career path, granting access to clinical and academic positions, as well as funding.

**Conclusions:**

Graduates from the MD-PhD program in Geneva were for the most part, satisfied with their training. However, because of the challenges and obstacles in combining clinical duties with research, the implementation of research activities in their current position proved difficult.

**Supplementary Information:**

The online version contains supplementary material available at 10.1186/s12909-020-02364-2.

## Background

A small proportion of all medical students and graduates are enrolled in MD-PhD programs, a degree program with the purpose of training research-oriented physicians. These highly skilled physicians, who undergo supplementary education in scientific research, are also known as ‘physician-scientists’. These physicians are expected to engage in biomedical research and academic careers, with the opportunity to play a significant role in medical education, research, and clinical practice of the future.

Since their establishment in the 1950’s, a growing number of MD-PhD programs are currently available in the United States [1]. However, in Europe, only a minority of medical academic institutions offer these degree programs which have only been in existence from the late 80’s and early 90’s [2–4]. In Switzerland, the national MD-PhD program was created in 1992 [4].

The characteristics, career intentions and outcomes of MD-PhD graduates have been thoroughly and periodically assessed in North America [5–11]. The available data supports attrition of physician-scientists after graduating from MD-PhD programs and there have been reports of restrictions in funding, which may be cause for concern [12–16]. It is possible that European physician-scientists face similar challenges to those reported in the US, but the literature on this matter is scarce [2, 4, 17–19]. Additionally, the structure of many European MD-PhD programs differs from that of the US, with various designs and candidature requirements [2]. Thus, there is a lack of detailed knowledge about the career outcomes of most European graduates of MD-PhD programs. In particular, the last available study on career outcomes of Swiss MD-PhD program graduates was published a decade ago [4].

Of note, in contrast to MD-PhD trainees in North America, a medical diploma is required to enroll into the MD-PhD program in Geneva. Therefore, the MD-PhD program in Geneva is a 3- to 5-year post-graduate scientific training, intended to empower medical doctors with applied research skills, whereas the program in the United States is a combined medical and PhD training.

The aim of the present study was to survey all physician-scientists who obtained the MD-PhD degree from the University of Geneva since 2010, using a questionnaire to assess demographic characteristics, research activity, career choices and also the challenges in combining research with clinical practice.

## Methods

### Ethical statement

Prior to its application, the design and the questionnaire of the present survey were reviewed by the Scientific Officer of the Dean’s office, Faculty of Medicine, University of Geneva. Voluntary participation and written consent to use the collected data were requested from all respondents. Confidentiality and anonymity of participants was ensured during both data collection and analysis.

### Study population

Eligible participants were defined as medical doctors who obtained their MD-PhD degree from the University of Geneva since 2010. According to the public alumni board from the University of Geneva accessed in April 2019, 32 graduates were included in the present study. One eligible participant was reported deceased during the study evaluation period and was excluded from the analysis.

### Study design

The present work is a descriptive cross-sectional study of the characteristics and outcomes of MD-PhD graduates from the University of Geneva between 2010 and 2019. All eligible participants received an online questionnaire via their institutional e-mail address in April 2019. One reminder was sent in May 2019.

### Questionnaire

In order to assess the study population, an anonymous 20-question online questionnaire (available in the online supplemental Table S1) was developed, based on previous studies [4, 6, 11]. The questionnaire was hosted on a Google survey platform and contained both objective and subjective questions. Closed-ended questions collected data regarding graduates’ demographics, MD-PhD training characteristics, career outcomes, publications, and opinions. Additionally, an open question was also included, to allow respondents the possibility of commenting on their experience with the program and any other related issues.

### Data collection and analysis

The study population did not require any sampling method because all eligible MD-PhD graduates were included. Response rate was calculated as the ratio between the number of respondents and the study population. Only completed questionnaires were assumed valid and included in the analyses. Data collected from respondents was summarized using descriptive statistics. Non-parametric tests were used for statistical inference, particularly the Mann-Whitney test when comparing two independent groups or Kruskal-Wallis test for higher number of groups. Associations between categorical variables were examined through Fisher’s exact test. Statistical analysis was performed in the R environment. All statistical tests were two-sided with a level of statistical significance set at *p* < 0.05.

## Results

Out of the 31 medical doctors invited to participate in the current survey, 21 replied to the questionnaire (response rate 65.5%). Demographic characteristics at enrolment in the survey are summarized in Table 1. Males represented 67% of survey respondents (14 out of 21), comparable to the overall population of MD-PhD graduates from the University of Geneva during the same period (71.9%, 23 out of 32). Regarding nationality, 57.1% of the MD-PhD graduates from the University of Geneva were Swiss citizens. 71.4% were still working in Switzerland at the time of the survey.

**Table 1:** Demographic characteristics of MD-PhD graduates in University of Geneva 2010–2019

Gender, n (%)	
Female	7 (33.3%)
Male	14 (66.7%)
Current country, n (%)	
Australia	1 (4.8%)
Saudi Arabia	2 (9.5%)
South Africa	1 (4.8%)
Switzerland	15 (71.4%)
USA	2 (9.5%)
Nationality, n (%)	
Cameroon	1 (4.8%)
France	3 (14.3%)
Italy	2 (9.5%)
Mexico	1 (4.8%)
Saudi Arabia	2 (9.5%)
Switzerland	12 (57.1%)
Marital status	
Married / with partner	5 (23.8%)
Married / with partner Parent	11 (52.4%)
Single	5 (23.8%)

**Fig. 1:**
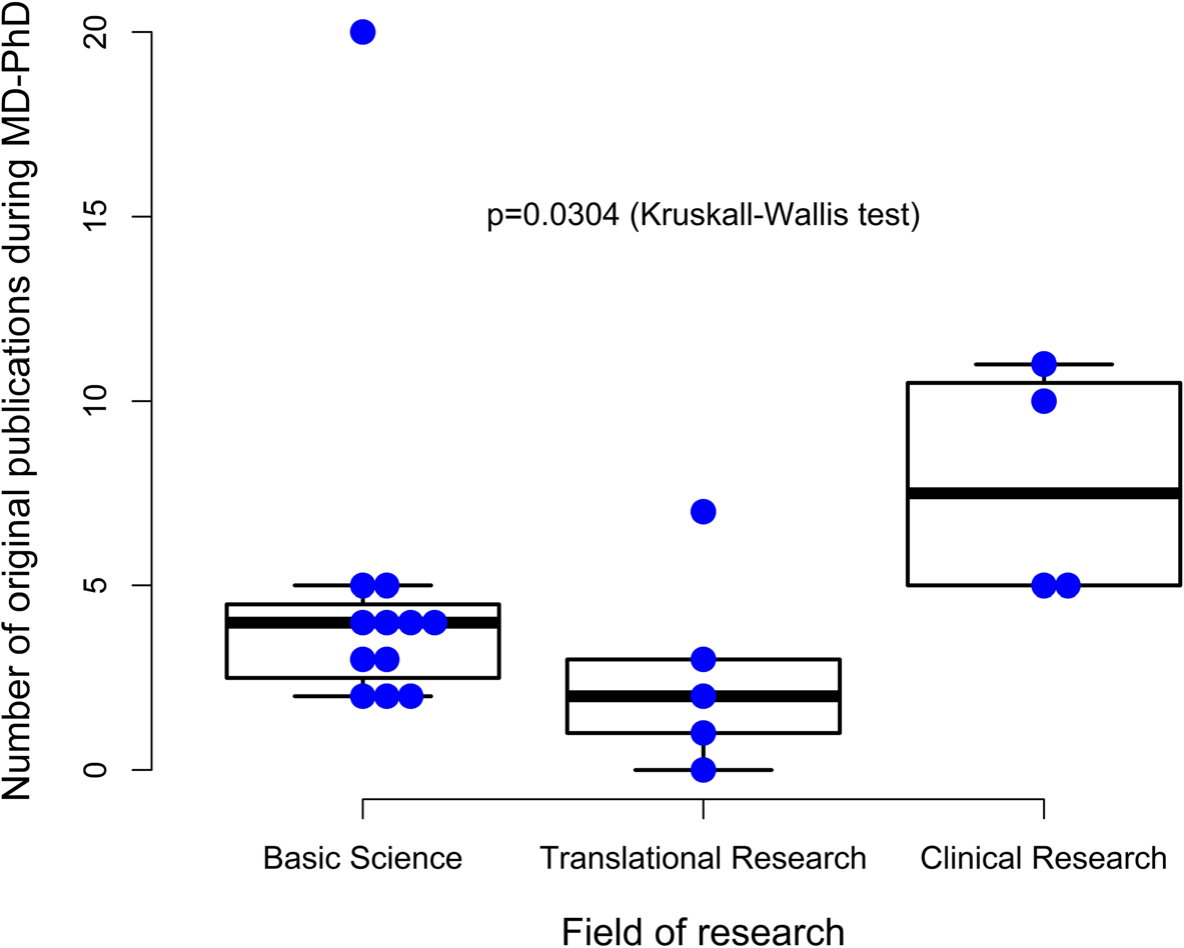
Number of original publications during the MD-PhD training, according to the type of research performed (basic, translational, clinical research)

Concerning the characteristics of those who had participated in MD-PhD training (Table 2), students had a relatively wide age-span by the time of their graduation (27 to 46 years) with their PhD training portion lasting typically three to 5 years. Students from the Geneva MD-PhD program published a median of four original articles during their degree training and there was evidence for a significant difference in the number of publications between the fields of research (basic science, translational research and clinical research. Figure 1, *p* = 0.03). Students who followed a clinical research training program, published a median of 7.5 original papers during MD-PhD training, whereas their peers in basic and translational research published a median of four and two original papers, respectively. There was no evidence for a significant difference in training length, graduation age, gender or funding concerning the three types of research (Table S2).

**Table 2:** MD-PhD program characteristics for MD-PhD graduates in University of Geneva 2010–2019

Age at time of MD-PhD graduation, years	
Mean (sd)	32 (4)
Median (min-max)	31 (27 to 46)
Field of MD-PhD research	
Basic science	12 (57.1%)
Clinical research	4 (19.0%)
Translational research	5 (23.8%)
MD-PhD training duration, n (%)	
3 years	9 (42.9%)
4 years (including 3.5 y)	7 (33.3%)
5 years	5 (23.8%)
MD-PhD funding, n (%)	
European grant	1 (4.8%)
Grant from host lab / institution	13 (61.9%)
Grant from SAMS^a^	6 (28.6%)
Grant SCES^b^	1 (4.8%)
Number of original papers during MD-PhD	
Mean (sd)	4.9 (4.4)
Median (min-max)	4 (0 to 20)

^a^*SAMS* Swiss Academy of Medical Sciences ^b^*SCES* Swiss Confederation Excellence Scholarship

The respondents had obtained the MD-PhD degree from the University of Geneva between one and 9 years prior to the survey (Table 3). During this time lapse, 57.1% of the respondents had already finished a medical specialty training, whereas 28.6% were in a residency training program. Since the MD-PhD graduation, the respondents had published a median of 1 (0 to 4.2) original articles per year. Of those who had reported carrying out any research activities at the time of the survey (71.4%), the publication rate was a median of two articles per year.

**Table 3:** Current professional situation of MD-PhD graduates 2010–2019

Time (in years) since MD-PhD graduation	
Mean (sd)	4.8 (2.9)
Median (min-max)	6 (1 to 9)
Number of original publications per year after MD-PhD	
Mean (sd)	1.5 (1.2)
Median (min-max)	1.0 (0.0 to 4.2)
Time dedicated to research, n (%)	
0%	6 (28.6%)
1–20%	6 (28.6%)
21–40%	1 (4.8%)
41–60%	3 (14.3%)
61–80%	2 (9.5%)
81–99%	1 (4.8%)
100%	2 (9.5%)
Current position, n (%)	
Peripheral hospital practitioner	1 (4.8%)
Private health care practitioner	1 (4.8%)
Research (either clinical or laboratory)	3 (14.3%)
University hospital - resident (interne)	6 (28.6%)
University hospital - senior registrar (chef de clinique)	6 (28.6%)
University hospital - consultant (médecin adjoint et plus)	4 (19%)

**Fig. 2:**
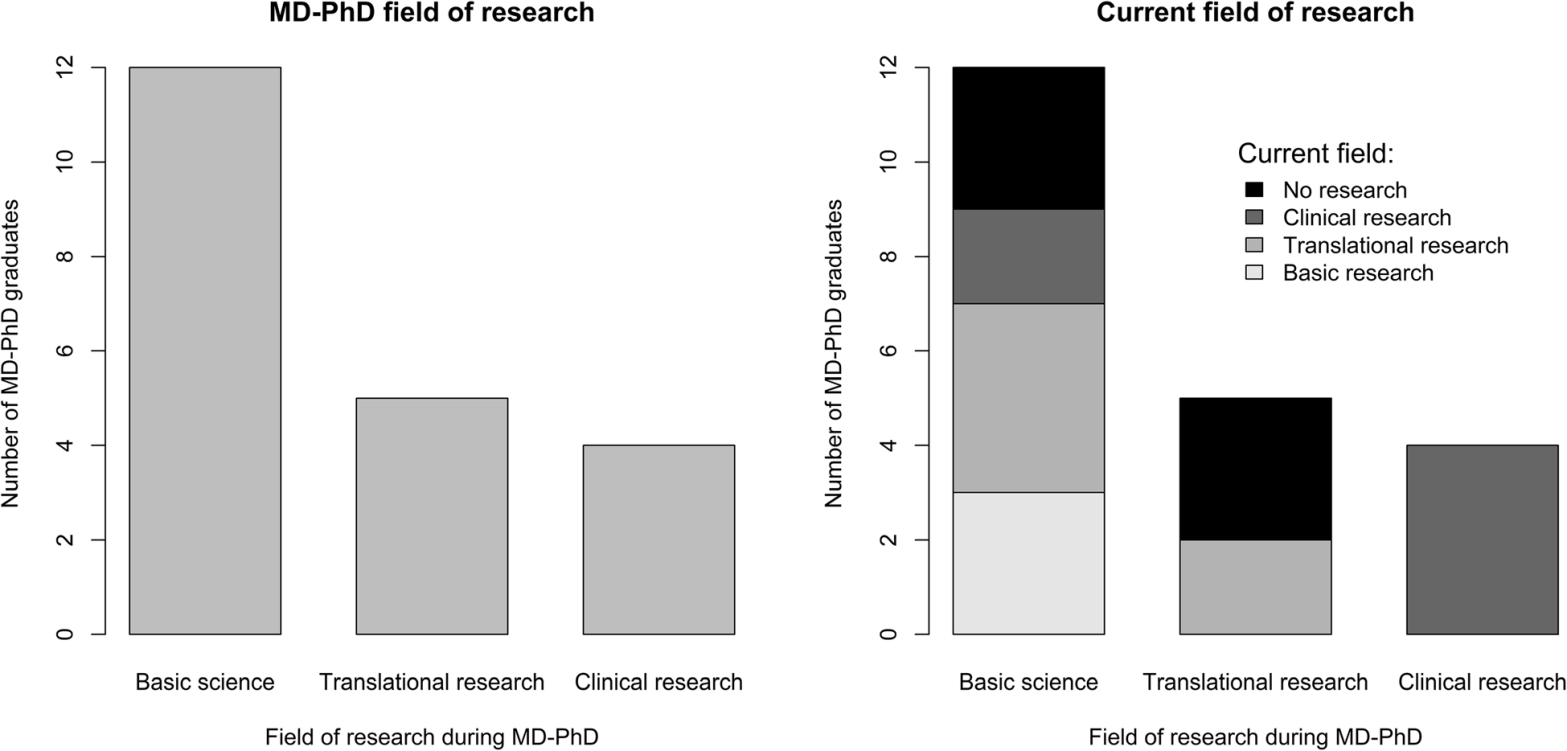
Field of research during MD-PhD training (left panel). Field of research in the current situation (right panel), represented in relation to the field of research during MD-PhD training

Overall, 28.6% of MD-PhD graduates from the University of Geneva were not involved in any research activity in their current work position. Despite this, by the time of the survey, most of the respondents were holding a position at a University hospital (90.5%), while only two were practicing in either private care or at a peripheral hospital (Table 3). Furthermore, of the MD-PhD graduates who completed their scientific training in basic sciences (12 out of 21), only three had pursued this type of research in their current position (Fig. 2). Finally, all four graduates who had undergone MD-PhD training in clinical research still had time allocated to clinical research in their current positions.

Data were also analyzed per gender (Table S3). The training duration, field of research and number of publications during the MD-PhD program were not significantly different between genders. However, the number of original publications per year after the MD-PhD graduation was significantly lower in female than in male respondents (0.3 vs 1.9, *p* = 0.022). This difference between females and males remained statistically significant in the subgroup of graduates who reported having allocated research time (1.0 vs 2.1, *p* = 0.032).

In examining the perspectives and opinions of MD-PhD graduates (Table 4), 85.7% considered that their degree had given them an advantage during their career, granting better clinical, academic or research positions, as well as the possibility of post-doctorate funding. However, 85.7% reported obstacles and challenges in combining clinical duties with research activities. The most prominent challenges seemed to be the lack of dedicated time set aside for research (61.1% of the respondents), an absence of mentoring (33.3%), under-compensation for the effort involved in producing quality research articles (27.8%), a lack of funding (33.3%) and difficulties in balancing family with work responsibilities (38.9%). Despite this, the MD-PhD graduates were for the most part satisfied with their training during the program at the University of Geneva and they would strongly recommend the MD-PhD program to a colleague who is interested in research.

**Table 4:** Perspectives and opinions of the MD-PhD graduates from the University of Geneva

Did your MD-PhD degree give you an edge during your career?	
No	3 (14.3%)
Yes	18 (85.7%)
If Yes (*n* = 18),	
Better clinical position	14 (77.8%)
Better academic or research position	13 (72.2%)
Better post-doc funding	4 (22.2%)
Are there any obstacles/challenges to combine clinical work and research?	
No	3 (14.3%)
Yes	18 (85.7%)
If Yes, what have been the most pressing obstacles/challenges?	
Lack of (dedicated) time	11 (61.1%)
Lack of mentoring	6 (33.3%)
Under-compensation	5 (27.8%)
Lack of funding	6 (33.3%)
Balancing family and work responsibilities	7 (38.9%)
Other^a^	7 (38.9%)
How satisfied are you with the MD-PhD programme? (0–10 scale)	
Mean (sd)	8.3 (1.5)
Median (min-max)	8 (4 to 10)
How strongly would you suggest the MD-PhD programme to a colleague/student that is interested in research? (0–10 scale)	
Mean (sd)	8.2 (2.1)
Median (min-max)	9 (1 to 10)
Would you do it again?	
No	1 (4.8%)
Yes	20 (95.2%)

^a^Other (Not finding position in desired location, Lack of opportunity, Satisfactory professional advancement)

## Discussion

The current survey shows that MD-PhD graduates from the University of Geneva who obtained their degree(s) in the decade 2010–2019 have very heterogeneous characteristics and career outcomes. In agreement with similar surveys of other physician-scientists’ populations [5], we observed a multi-national profile amongst graduates. Considering the wide age-span by the time of MD-PhD graduation, some of the differences noted can be attributed to trainees being at different stages of their professional and personal career. Nevertheless, we also identified common aspects, challenges, and career choices between the respondents.

MD-PhD students participated in a variety of different types of research projects during their training. Our data shows that 57.1% pursued scientific training in fundamental sciences, yet only 14.3% pursued this type of research in their current positions. Considering our results, the MD-PhD pathway in clinical research was associated with a higher number of original publications during MD-PhD training. This aspect may be important to candidates, mentors, and policy makers, in order to tailor MD-PhD programs to better encompass post graduate career planning. Likewise, increased funding and institutional strategies may be required to finance more dedicated research time for MD-PhD graduates in the fundamental sciences.

It is important to note that 28.6% of the MD-PhD graduates mentioned not having any allocated research time in their current position. Most of these were young MD-PhD graduates with full-time clinical duties at the University hospital. However, it also included graduates who had left this environment in order to pursue a career in a peripheral hospital or the private care sector. Similarly, studies from the United States have found that 14–16% of MD-PhD graduates do not pursue research careers [5, 10]. Taking into consideration that the lack of allocated research time was the main obstacle acknowledged by our respondents, it may be necessary for hospital policy makers to allocate specific research time to young physician-scientists, in order to ensure a vigorous and vibrant research environment for the future and to avoid high attrition rates amongst the MD-PhD workforce.

In addition to the insufficient research time that was mentioned by 61.1% of respondents, about a third of MD-PhD graduates also faced lack of mentoring or lack of funding. 27.8% expressed a sentiment of under-compensation and 38.9% mentioned difficulties balancing family with work responsibilities. Indeed, sub-optimal supervision and mentoring had already been reported in a Swiss survey analyzing MD-PhD graduate outcomes between 1992 and 2007 [4]. More recently, since 2017, the Swiss Academy of Medical Sciences, in collaboration with the Gottfried and Julia Bangerter-Rhyner Foundation have been supporting research by young medical doctors during their residency, with the *Young Talents in Clinical Research (YTCR)* program [20], to help counteract the scarcity of time and funding. Other actions have been taken in recent years to help under- and postgraduates who lack supervision and mentoring. Several mentoring programs are available at the University of Geneva [21–23] to provide guidance during the academic career and facilitate the transition after graduation.

Despite the reported challenges, almost all graduates (85.7%) considered that this degree was beneficial to their career and were satisfied with the program in Geneva. As a result, graduates highly recommended this program for future newcomers and 95.2% replied that, faced with the same choices, they would take the program again.

This survey which collected data on graduates spanning 10 years revealed that females represented only 28.9% of MD-PhD graduates from the University of Geneva. Accordingly, a Swiss survey analyzing MD-PhD program outcomes between 1992 and 2007 reported 23% of women finished the program [4]. Additionally, in our study, female physician-scientists had a lower rate of publications per year after the MD-PhD degree compared to their male counterparts. Noteworthy, somewhat similar findings are reported in the United States [24] and Canada [7], where female MD-PhD graduates were less likely to be funded and had lesser sustained research involvement. A possible reason for this finding is that balancing family life with work responsibilities may be a harder challenge for young female researchers in comparison to their male peers. Although these discrepancies are currently being addressed [25], it is important to realize the challenges involved in providing equal opportunities in the MD-PhD career setting.

Even though there is lack of data to compare the MD-PhD program in Geneva with that used in the USA (combined medical and PhD training), this study was still insightful in that the training format entails completing PhD years after one’s medical training. This format is also being used in the US at certain programs, such as the UCLA STAR Program [26]. As such, it is debatable whether it would be best to do the PhD in a combined MD-PhD program or after graduating medical school during post graduate training. Despite these two possible tracks, similar issues with funding, having protected research time and work life balance are still major challenges. Given that funding levels and structures are different between the USA and Europe, direct comparisons would be limited.

We did not carry out an in depth assessment of the scientific impact of the MD-PhD program by collecting data on the impact factor of journals accepting the articles and/or the number of respective quotations from each published work. Thus, the number of original papers serves only as an estimation of the scientific output of the surveyed MD-PhD graduates.

The main limitation of this study is the small sample size. For this single-center study that surveyed graduates from the past 10 years, there were 31 eligible MD-PhD graduates. Despite this, we obtained a response rate of two-thirds of the surveyed population. Moreover, the respondents had a wide distribution in terms of age, and they had the same distribution by gender compared to the total surveyed population; thus, this should be a representative sample. This, in turn, should limit the risks of biased conclusions based on the obtained responses. Therefore, we expect our data to accurately represent the situation of most of the MD-PhD graduates from the University of Geneva. Due to the small sample size, we did not perform regression analysis to adjust for potential confounders and the statistical tests are of limited power. Hence, generalizations to other physician-scientists populations based on our dataset should be cautious.

This survey of 10 years’ worth of graduates brings valuable information in an area that suffers from a paucity of literature. As shown by previous studies [27], the assessment of individuals following MD-PhD training and the recognition of the challenges graduates face may favor future improvements. Furthermore, such challenges appear to be similar whether that be in the United States or in Europe. Hospital policy makers may need to address the issues brought up in this article so that that the future of research carried out by medical doctors working in the clinical setting can be ensured.

## Conclusion

Among MD-PhD graduates of the University of Geneva, we identified a high satisfaction rate and a successful scientific career profile. However, among the MD-PhD graduate population in Geneva there are gender discrepancies and career challenges in combining research with clinical duties in a university hospital setting.

The continuous assessment and improvement of MD-PhD programs is of paramount importance as these physicians are a valuable resource in biomedical research, allowing for the translation of biomedical knowledge from the bench to the bedside. Their unique training provides the clinical understanding and the necessary skills required to develop innovative and relevant research that can directly impact patient care and treatment options.

## Supplementary Information

**Additional file 1: Table S1.** - Questionnaire for MD-PhD graduates from *Université de Genève*. **Table S2.** MD-PhD program characteristics and outcomes, considering the different fields of MD-PhD research. **Table S3.** Characteristics and outcomes of MD-PhD graduates, considering gender. **FIGURE S1** – Number of publications during MD-PhD training (left), represented by gender. On the right, number of publications per year since MD-PhD graduation, represented by gender.

## Data Availability

The datasets used during the current study are available from the corresponding author on reasonable request.

## References

[1] Harding CV, Akabas MH, Andersen OS (2017). History and outcomes of 50 years of physician-scientist training in medical scientist training programs. Acad Med.

[2] Scherlinger M, Bienvenu TCM, Piffoux M, Séguin P (2018). Les doubles cursus médecine-sciences en France: État des lieux et perspectives. médecine/sciences.

[3] Barnett-Vanes A, Ho G, Cox TM (2015). Clinician-scientist MB/PhD training in the UK: a nationwide survey of medical school policy. BMJ Open.

[4] Kuehnle K, Winkler DT, Meier-Abt PJ (2009). Swiss national MD-PhD-program: an outcome analysis. Swiss Med Wkly.

[5] Andriole DA, Whelan AJ, Jeffe DB (2008). Characteristics and career intentions of the emerging MD/PhD workforce. Jama.

[6] Kwan JM, Daye D, Schmidt ML, Conlon CM, Kim H, Gaonkar B (2017). Exploring intentions of physician-scientist trainees: factors influencing MD and MD/PhD interest in research careers. BMC Med Educ.

[7] Skinnider MA, Twa DDW, Squair JW, Rosenblum ND, Lukac CD (2018). Predictors of sustained research involvement among MD/PhD programme graduates. Med Educ.

[8] Jeffe DB, Andriole DA, Wathington HD, Tai RH (2014). The emerging physician-scientist workforce: demographic, experiential, and attitudinal predictors of MD-PhD program enrollment. Acad Med.

[9] Jeffe DB, Andriole DA, Wathington HD, Tai RH (2014). Educational outcomes for students enrolled in MD-PhD programs at medical school matriculation, 1995-2000: a national cohort study. Acad Med.

[10] Brass LF, Akabas MH, Burnley LD, Engman DM, Wiley CA, Andersen OS (2010). Are MD-PhD programs meeting their goals? An analysis of career choices made by graduates of 24 MD-PhD programs. Acad Med.

[11] Skinnider MA, Squair JW, Twa DDW, Ji JX, Kuzyk A, Wang X (2017). Characteristics and outcomes of Canadian MD/PhD program graduates: a cross-sectional survey. CMAJ Open.

[12] Jain MK, Cheung VG, Utz PJ, Kobilka BK, Yamada T, Lefkowitz R (2019). Saving the endangered physician-scientist - a plan for accelerating medical breakthroughs. N Engl J Med.

[13] Furuya H, Brenner D, Rosser CJ (2017). On the brink of extinction: the future of translational physician-scientists in the United States. J Transl Med.

[14] Kosik RO, Tran DT, Fan AP, Mandell GA, Tarng DC, Hsu HS (2016). Physician scientist training in the United States: a survey of the current literature. Eval Health Prof.

[15] Twa DD, Squair JW, Skinnider MA, Ji JX (2015). The Canadian clinician-scientist training program must be reinstated. J Clin Invest.

[16] Milewicz DM, Lorenz RG, Dermody TS, Brass LF (2015). Rescuing the physician-scientist workforce: the time for action is now. J Clin Invest.

[17] Spaniol K, Geerling G (2015). MD-PhD Programme. Wege zu einer grundlagenwissenschaftlichen Ausbildung für Augenärzte. Ophthalmologe.

[18] Gompels LL, Chinoy H, Devakumar V, Bax D, Mackworth-Young CG (2011). Academic training in rheumatology in 2009: a UK trainee survey. Clin Med (Lond).

[19] Radiology ESo (2013). MD PhD programmes with relevance to imaging. Results from a European survey. Insights Imaging.

[20] Young Talents in Clinical Research: Swiss Academy Medical Sciences; 2017–2020 [encouraging young medical doctors to venture into clinical research]. Available from: https://www.samw.ch/en/Funding/Young-Talents-Clinical-Research.html. Accessed 06 Oct 2020.

[21] Faculty of Medicine mentoring programme - University of Geneva [Available from: https://www.unige.ch/medecine/mentoring/. Accessed 09 Oct 2020.

[22] University of Geneva - Mentorat [Available from: https://www.unige.ch/dife/carriere/mentorat1/. Accessed 09 Oct 2020.

[23] Mentoring Program For Postdocs Of The University Of Geneva [Available from: https://www.unige.ch/apdu/mentoring-program/. Accessed 09 Oct 2020.

[24] Ley TJ, Hamilton BH (2008). Sociology. The gender gap in NIH grant applications. Science..

[25] Andriole DA, Jeffe DB (2016). Predictors of full-time faculty appointment among MD-PhD program graduates: a national cohort study. Med Educ Online.

[26] Medical Education Research with the UCLA STAR Program: UCLA David Geffen School of Medicine; since 1993 [Available from: https://medschool.ucla.edu/star-about-star.

[27] Gotian R, Raymore JC, Rhooms SK, Liberman L, Andersen OS (2017). Gateways to the laboratory: how an MD-PhD program increased the number of minority physician-scientists. Acad Med.

