# Supplementary material for: The MD-PhD program in Geneva: a 10-year analysis of graduate demographics and outcomes
Source: BMC Med Educ. 2020 Nov 12;20:425. doi: 10.1186/s12909-020-02364-2 (PMC7663876; doi:10.1186/s12909-020-02364-2)

***- ONLINE SUPPLEMENTAL DATA –***

THE MD-PhD PROGRAM IN GENEVA: A 10-YEAR ANALYSIS OF GRADUATE DEMOGRAPHICS AND OUTCOMES

Andre Dos Santos Rocha^1,2^, Cristophe Combescure^3^, Francesco Negro^4,5^

^1^ Unit for Anaesthesiological Investigations, Department of Acute Medicine, University of Geneva, Switzerland

^2^ European MD-PhD Association, Groningen, The Netherlands

^3^ Division of Clinical Epidemiology, University Hospitals of Geneva, Switzerland

^4^ MD-PhD Committee, Faculty of Medicine, University of Geneva, Switzerland

^5^ Divisions of Gastroenterology and Hepatology and of Clinical Pathology, University Hospitals of Geneva, Geneva, Switzerland.

ADDRESS FOR CORRESPONDENCE:

Andre Dos Santos Rocha, MD

Unit for Anaesthesiological Investigations

Rue Michel-Servet 1, 1206 Genève, Switzerland

Phone: +41 (0)79 553 21 36

Email: [Andre.DosSantosRocha@unige.ch](mailto:Andre.DosSantosRocha@unige.ch)

**TABLE S1** - Questionnaire for MD-PhD graduates from *Université de Genève*

| 1. Please specify your age: |
| --- |
|  |
| 1. Gender: Male / Female |
|  |
| 1. What is your current country of work or training? |
|  |
| 1. What is your marital status? |
| - 1. Single |
| - 1. Single Parent |
| - 1. Married/With Partner |
| - 1. Married/With Partner Parent |
| - 1. Do not want to answer |
|  |
| 1. When did you obtained your MD-PhD degree? |
|  |
| 1. How long did your MD-PhD training last? |
| - 1. 3 years |
| - 1. 3 ½ years |
| - 1. 4 years |
| - 1. 4 ½ years |
| - 1. 5 years |
| - 1. Other: |
|  |
| 1. In which field did you perform your MD-PhD research? |
| - 1. Basic science |
| - 1. Translational research |
| - 1. Clinical research |
|  |
| 1. How was your MD-PhD training funded? |
| - 1. Grant from Swiss National Science Foundation (SNSF) |
| - 1. Grant from Host lab/institution |
| - 1. Grant from private Institute / Foundation |
| - 1. Other: |
|  |
| 1. Have you completed a specialty training (FMH specialist) - Yes / No? |
|  |
| 1. What position(s) do you currently have? (please select several options) |
| - 1. University hospital – resident (*Interne*) |
| - 1. University hospital - Senior Registrar (*Chef de clinique*) |
| - 1. University hospital – Consultant (*Médecin adjoint et plus*) |
| - 1. Professor at Swiss University |
| - 1. Professor at foreign University |
| - 1. Peripheral hospital practitioner |
| - 1. Private practice practitioner |
| - 1. Researcher (either clinical or laboratory) |
| - 1. FMH specialist |
| - 1. University teaching (without professorship) |
| - 1. Industry |
| - 1. Public health policy |
| - 1. Non-medical (consultancy/business, journalism, politics etc.) |
|  |
| 1. How many original papers did you publish during your MD-PhD studies? |
|  |
| 1. How many original papers did you publish since you obtained the MD-PhD degree? |
|  |
| 1. Do you consider that your MD-PhD degree gave you an edge during your career? |
| - 1. Yes, it granted me access to better clinical positions |
| - 1. Yes, it granted me access to better research positions |
| - 1. Yes, it granted me access to better academic positions |
| - 1. Yes, it granted me access to post-doc funding |
| - 1. No, it gave me no advantage |
|  |
| 1. How much of your working time is currently devoted to research? |
| - 1. 0% |
| - 1. 0-20% |
| - 1. 20-40% |
| - 1. 40-60% |
| - 1. 60-80% |
| - 1. 100% |
|  |
| 1. What is the nature of the research you are doing? |
| - 1. Clinical |
| - 1. Basic Science |
| - 1. Translational |
| - 1. Public Health/Policy Making |
| - 1. Other: |
| - 1. N/A |
|  |
| 1. What have been the three most pressing obstacles/challenges to combine clinical work and research? |
| - 1. None |
| - 1. Lack of (protected) time |
| - 1. Lack of mentoring |
| - 1. Under-compensation |
| - 1. Lack of funding |
| - 1. Lack of opportunity |
| - 1. Not finding position in desired location |
| - 1. Balancing family and work responsibilities |
| - 1. Satisfactory professional advancement |
| - 1. Discrimination against your gender/ethnicity/sexual orientation |
| - 1. Other: |
|  |
| 1. How satisfied are you with the MD-PhD programme in *Université de Genève*? (scale 1-10) |
|  |
| 1. How strongly would you suggest the MD-PhD programme in *Université de Genève* to a colleague/student that is interested in research? (scale 1-10) |
|  |
| 1. Would you do it again? - Yes / No? |
|  |
| 1. Are there any other comments that you would like to give? |

**TABLE S2**: MD-PhD program characteristics and outcomes, considering the different fields of MD-PhD research

|  | Field of MD-PhD research | | |  |
| --- | --- | --- | --- | --- |
|  | Basic science | Clinical research | Translational research |  |
| n | 12 | 4 | 5 | p=0.0304 (Kruskal-Wallis test) |
| Number of publications during MD-PhD |  |  |  |  |
| Median (min-max) | 4.0 (2.0 to 20.0) | 7.5 (5.0 to 11.0) | 2.0 (0.0 to 7.0) |  |
| MD-PhD duration, n (%) |  |  |  | p=0.66 (Fisher exact test) |
| 3 years | 5 (41.7%) | 2 (50.0%) | 2 (40.0%) |  |
| 4 years (including 3.5 y) | 4 (33.3%) | 2 (50.0%) | 1 (20.0%) |  |
| 5 years | 3 (25.0%) | 0 (0.0%) | 2 (40.0%) |  |
| Gender, n (%) |  |  |  | p=0.57 (Fisher exact test) |
| Female | 3 (25.0%) | 2 (50.0%) | 2 (40.0%) |  |
| Male | 9 (75.0%) | 2 (50.0%) | 3 (60.0%) |  |
| Age at time of MD-PhD graduation, years |  |  |  | p=0.14 (Kruskal-Wallis test) |
| Median (min-max) | 31 (27 to 35) | 35 (31 to 46) | 30 (27 to 36) |  |
| MD-PhD funding, n (%) |  |  |  | p=0.53 (Fisher exact test) |
| European grant | 1 (8.3%) | 0 (0.0%) | 0 (0.0%) |  |
| Grant from host lab / institution | 7 (58.3%) | 3 (75.0%) | 3 (60.0%) |  |
| Grant from SNSF* | 4 (33.3%) | 0 (0.0%) | 2 (40.0%) |  |
| Grant from SCES** | 1 (25.0%) | 0 (0.0%) | 2 (40.0%) |  |
|  |  | | |  |
| Current field of research |  |  |  |  |
| Basic science | 3 (25.0%) | 0 (0.0%) | 0 (0.0%) |  |
| Clinical research | 2 (16.7%) | 4 (100.0%) | 0 (0.0%) |  |
| Translational research | 4 (33.3%) | 0 (0.0%) | 2 (20.0%) |  |
| No reasearch activity | 3 (25.0%) | 0 (0.0%) | 3 (60.0%) |  |
| Current position, n (%) |  |  |  |  |
| Peripheral hospital practitioner | 1 (8.3%) | 0 (0%) | 0 (0%) |  |
| Private health care practitioner | 1 (8.3%) | 0 (0%) | 0 (0%) |  |
| Research (either clinical or laboratory) | 1 (8.3%) | 2 (50%) | 0 (0%) |  |
| University hospital - resident (interne) | 3 (25%) | 0 (0%) | 3 (60%) |  |
| University hospital - senior registrar (chef de clinique) | 3 (25%) | 1 (25%) | 1 (20%) |  |
| University hospital - subspeciality fellow | 1 (8.3%) | 0 (0%) | 0 (0%) |  |
| University hospital - consultant (médecin adjoint et plus) | 2 (16.7%) | 1 (25%) | 1 (20%) |  |
| Time dedicated to research in current position, n (%) | |  |  |  |
| 0% | 3 (25.0%) | 0 (0.0%) | 3 (60.0%) |  |
| 1-20% | 4 (33.3%) | 1 (25.0%) | 1 (20.0%) |  |
| 21-40% | 1 (8.3%) | 0 (0.0%) | 0 (0.0%) |  |
| 41-60% | 1 (8.3%) | 1 (25.0%) | 1 (20.0%) |  |
| 61-80% | 1 (8.3%) | 1 (25.0%) | 0 (0.0%) |  |
| 81-99% | 1 (8.3%) | 0 (0.0%) | 0 (0.0%) |  |
| 100% | 1 (8.3%) | 1 (25.0%) | 0 (0.0%) |  |
| Field of research research in persons with at least 1% of time dedicated  to research | | |  |  |
| Basic science | 3 (33.3%) | 0 (0.0%) | 0 (0.0%) |  |
| Clinical research | 2 (22.2%) | 4 (100.0%) | 0 (0.0%) |  |
| Translational research | 4 (44.4%) | 0 (0.0%) | 2 (100.0%) |  |

*SNSF: Swiss National Science Foundation **SCES: Swiss Confederation Excellence Scholarship

**TABLE S3**: Characteristics and outcomes of MD-PhD graduates, considering gender.

|  | **Female (n=7)** | **Male (n=14)** |  |
| --- | --- | --- | --- |
| Field MD-PhD |  |  |  |
| Basic science | 3 (42.9%) | 9 (64.3%) |  |
| Clinical research | 2 (28.6%) | 2 (14.3%) |  |
| Translational research | 2 (28.6%) | 3 (21.4%) |  |
| MD-PhD duration, n (%) |  |  | p=0.64 (Fisher exact test) |
| 3 years | 2 (28.6%) | 7 (50%) |  |
| 4 years (including 3.5 y) | 2 (28.6%) | 5 (35.7%) |  |
| 5 years | 3 (42.9%) | 2 (14.3%) |  |
| Number of publications during MD-PhD |  |  | p=0.82 (Mann-Whitney test) |
| Median (min-max) | 4.0 (1.0 to 11.0) | 4.0 (0.0 to 20.0) |  |
| Current position |  |  |  |
| Peripheral hospital practitioner | 0 (0.0%) | 1 (7.1%) |  |
| Private health care practitioner | 0 (0.0%) | 1 (7.1%) |  |
| Research (either clinical or laboratory) | 2 (28.6%) | 1 (7.1%) |  |
| University hospital - resident (interne) | 2 (28.6%) | 4 (28.6%) |  |
| University hospital - senior registrar (chef de clinique) | 1 (14.3%) | 4 (28.6%) |  |
| University hospital - subspeciality fellow | 1 (14.3%) | 0 (0.0%) |  |
| University hospital - consultant (médecin adjoint et plus) | 1 (14.3%) | 3 (21.4%) |  |
| Time dedicated to research in the current position, n (%) |  |  |  |
| 0% | 2 (28.6%) | 4 (28.6%) |  |
| 1-20% | 1 (14.3%) | 5 (35.7%) |  |
| 21-40% | 0 (0%) | 1 (7.1%) |  |
| 41-60% | 1 (14.3%) | 2 (14.3%) |  |
| 61-80% | 1 (14.3%) | 1 (7.1%) |  |
| 81-99% | 1 (14.3%) | 0 (0%) |  |
| 100% | 1 (14.3%) | 1 (7.1%) |  |
| Number of publications per year after MD-PhD |  |  | p=0.0221 (Mann-Whitney test) |
| Median (min-max) | 0.3 (0.0 to 2.0) | 1.9 (0.4 to 4.2) |  |
| Number of publications per year after MD-PhD in persons with at least 1% of time dedicated to research (n=15) |  |  | p=0.0316 (Mann-Whitney test) |
| n | 5 | 10 |  |
| Median (min-max) | 1.0 (0.0 to 2.0) | 2.1 (0.7 to 4.2) |  |
| Do you consider that your MD-PhD degree gave you an edge during your career? |  |  |  |
| No | 1 (14.3%) | 2 (14.3%) |  |
| Yes | 6 (85.7%) | 12 (85.7%) |  |
| If Yes (n=18), |  |  |  |
| n | 6 | 12 |  |
| Better clinical position | 4 (66.7%) | 10 (83.3%) |  |
| Better academic or research position | 5 (83.3%) | 8 (66.7%) |  |
| Better post-doc funding | 2 (33.3%) | 2 (16.7%) |  |
| Are there any pressing obstacles/challenges to combine clinical work and research? |  |  |  |
| No | 1 (14.3%) | 2 (14.3%) |  |
| Yes | 6 (85.7%) | 12 (85.7%) |  |
| If Yes, what have been the most pressing obstacles/challenges? |  |  |  |
| n | 6 | 12 |  |
| Lack of time | 4 (66.7%) | 7 (58.3%) |  |
| Lack of mentoring | 2 (33.3%) | 4 (33.3%) |  |
| Under compensation | 0 (0.0%) | 5 (41.7%) |  |
| Lack of funding | 2 (33.3%) | 4 (33.3%) |  |
| Balance with family | 3 (50.0%) | 4 (33.3%) |  |
| Other | 3 (50.0%) | 4 (33.3%) |  |
| How satisfied are you with the MD-PhD programme ? (0-10 scale) |  |  |  |
| Median (min-max) | 8.0 (7.0 to 10.0) | 8.5 (4.0 to 10.0) |  |
| How strongly would you suggest the MD-PhD programme to a colleague/student that is interested in research? (0-10 scale) |  |  |  |
| Median (min-max) | 8.0 (8.0 to 10.0) | 9.0 (1.0 to 10.0) |  |

**FIGURE S1** – Number of publications during MD-PhD training (left), represented by gender. On the right, number of publications per year since MD-PhD graduation, represented by gender.

*p* = 0.022 (Mann-Whitney test)


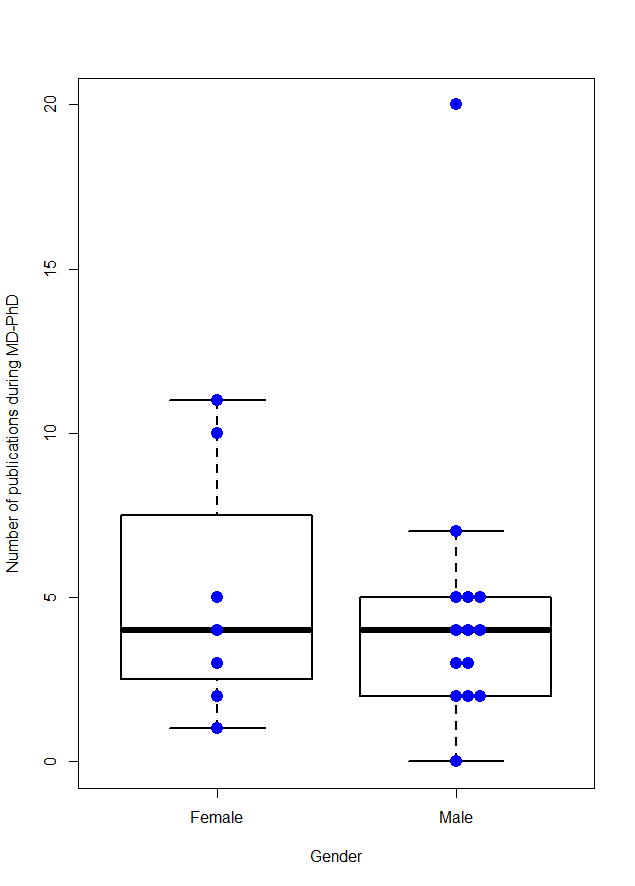

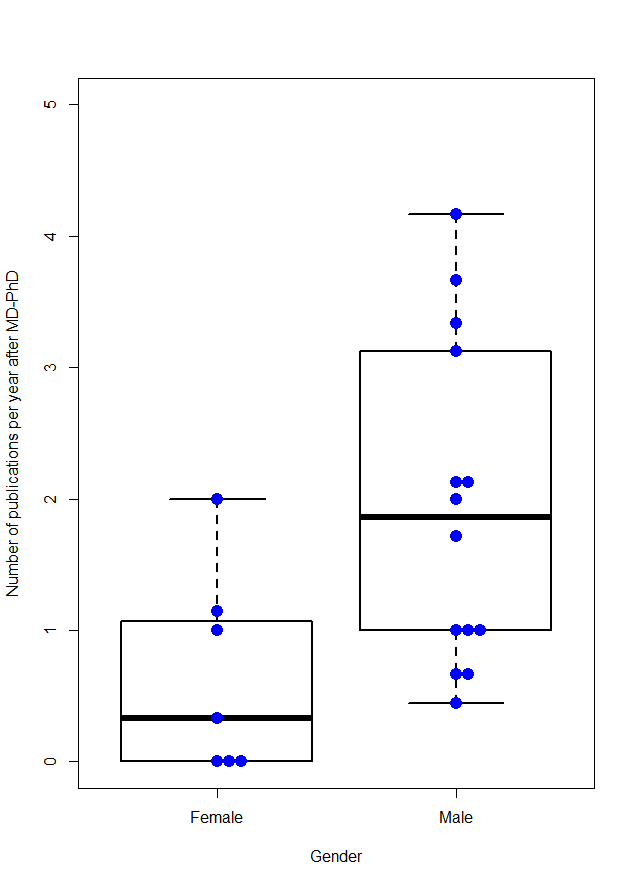

Supplement: Supplementary file 1 — Additional file 1: Table S1. - Questionnaire for MD-PhD graduates from Université de Genève. Table S2. MD-PhD program characteristics and outcomes, considering the different fields of MD-PhD research. Table S3. Characteristics and outcomes of MD-PhD graduates, considering gender. FIGURE S1 – Number of publications during MD-PhD training (left), represented by gender. On the right, number of publications per year since MD-PhD graduation, represented by gender. [file 12909_2020_2364_MOESM1_ESM.docx]
